# Supplementary material for: Splice-Junction-Based Mapping of Alternative Isoforms in the Human Proteome
Source: Cell Rep. Author manuscript; Available in PMC 2020 Jan 15. (PMC6961840; doi:10.1016/j.celrep.2019.11.026)
Supplement: 2 [file NIHMS1546469-supplement-2.zip › DF1/PXD006675/AorticValve/AorticValve_5_HDAC10_WFPELHLGLGAAPGLWLPADMTATR.pdf]

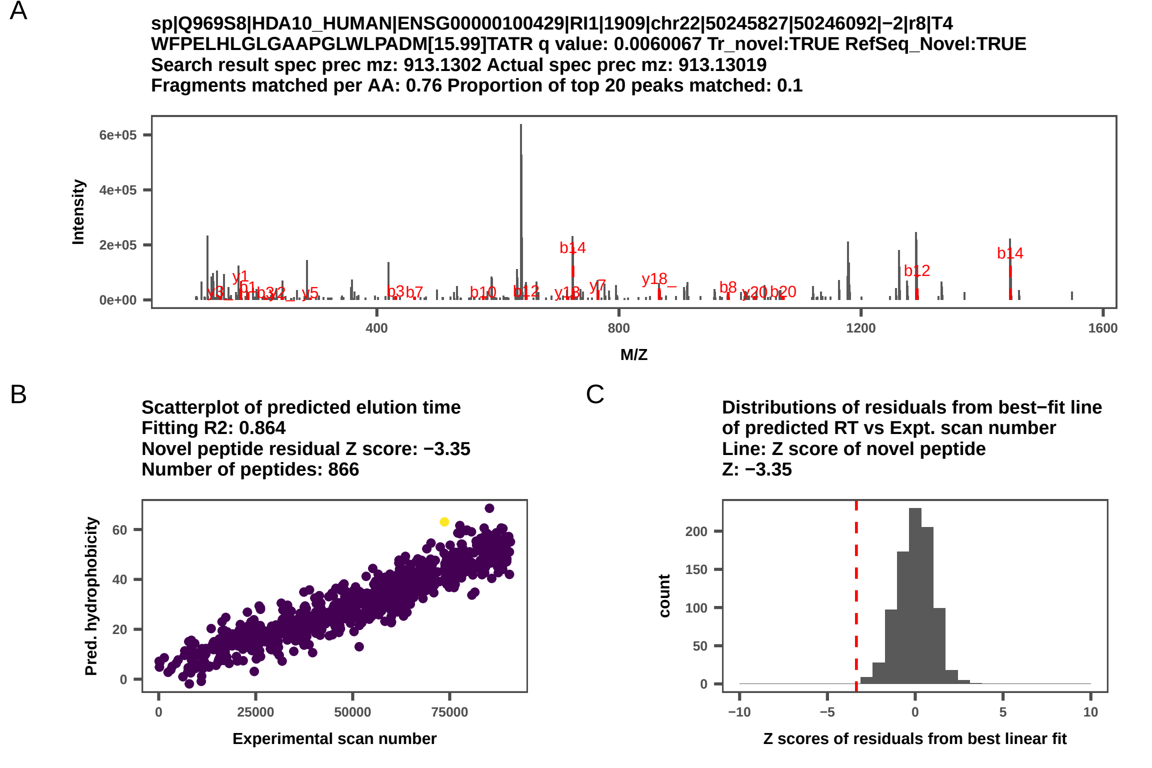
A
sp|Q969S8|HDA10_HUMAN|ENSG00000100429|RI1|1909|chr22|50245827|50246092|−2|r8|T4
WFPELHLGLGAAPGLWLPADM[15.99]TATR q value: 0.0060067 Tr_novel:TRUE RefSeq_Novel:TRUE
Search result spec prec mz: 913.1302 Actual spec prec mz: 913.13019
Fragments matched per AA: 0.76 Proportion of top 20 peaks matched: 0.1
Intensity
6e+05
4e+05
2e+05
0e+00
M/Z
400
800
1200
1600
y1
b14
b12
b14
y18_
y7
b8
y20
b20
b3
b7
b10
b12
y13
y5
B
Scatterplot of predicted elution time
Fitting R2: 0.864
Novel peptide residual Z score: −3.35
Number of peptides: 866
Pred. hydrophobicity
0
20
40
60
0
25000
50000
75000
Experimental scan number
C
Distributions of residuals from best−fit line
of predicted RT vs Expt. scan number
Line: Z score of novel peptide
Z: −3.35
count
0
50
100
150
200
−10
−5
0
5
10
Z scores of residuals from best linear fit
